# Stress-induced hyperglycemia is associated with the mortality of thrombotic thrombocytopenic purpura patients

**DOI:** 10.1186/s13098-024-01275-2

**Published:** 2024-02-15

**Authors:** Lingling Hu, Jing Wang, Xiaxia Jin, Guoguang Lu, Meidan Fang, Jian Shen, Tao-Hsin Tung, Bo Shen

**Affiliations:** 1 Department of Clinical Laboratory, Taizhou Hospital of Zhejiang Province Affiliated to Wenzhou Medical University, 150 Ximen Road, Linhai, Taizhou, Zhejiang Province China; 2 Key Laboratory of System Medicine and Precision Diagnosis and Treatment of Taizhou, Taizhou, China; 3 Evidence-Based Medicine Center, Taizhou Hospital of Zhejiang Province Affiliated to Wenzhou Medical University, 150 Ximen Road, Linhai, Taizhou, Zhejiang Province China; 4 Department of Hematology, Taizhou Hospital of Zhejiang Province Affiliated to Wenzhou Medical University, 150 Ximen Road, Linhai, Taizhou, Zhejiang Province China

**Keywords:** Thrombotic thrombocytopenic purpura, Prognosis, Platelet, Lactate dehydrogenase

## Abstract

**Background:**

Thrombotic thrombocytopenic purpura (TTP) is a rare thrombotic microangiopathy with a rapid progression and high mortality rate. We aimed to explore early risk factors for mortality in patients with TTP.

**Methods:**

We conducted a retrospective analysis of 42 TTP patients that were admitted to our hospital between 2000 and 2021, with a median age of 49 (29–63) years. Risk factors for mortality were evaluated using multivariate logistic regression. Receiver operating characteristic curve analysis was used to determine the cut-off value of glucose for predicting mortality in patients, which was validated by comparison to a similar cohort in the published literature.

**Results:**

Elevated glucose level and reduced red blood cells (RBC) counts were risk factors for mortality in patients with TTP (glucose, odds ratio and 95% confidence interval: 2.476 [1.368–4.484]; RBC, odds ratio and 95% confidence interval: 0.095 [0.011–0.799]). The area under the curve of glucose was 0.827, and the cut-off value was 9.2 mmol/L, with a sensitivity of 75.0% and specificity of 95.8%. A total of 26 cases from the validation cohort had a sensitivity of 71.0% and a specificity of 84.0%. The change trends of the TTP-related laboratory indices differed during hospitalization.

**Conclusion:**

Hyperglycemia at admission and unstable blood glucose levels during hospitalization may be potential predictors of mortality for TTP patients. The improved prognosis was associated with the recovery of platelet counts and a significant decrease in serum lactate dehydrogenase after five days of treatment.

**Supplementary Information:**

The online version contains supplementary material available at 10.1186/s13098-024-01275-2.

## Introduction

Thrombotic thrombocytopenic purpura (TTP) is a rare thrombotic microangiopathy with a global annual incidence of approximately 1.5–6 per million people and a female predominance [1]. It has a rapid progression with a mortality rate of untreated TTP as high as 90%; this can be reduced to 10 − 20% by early identification, plasma exchange, and immunosuppressive therapy [2, 3]. TTP is characterized by microangiopathic hemolytic anemia (MAHA), thrombocytopenia, and neurologic deficits (the triad of TTP symptoms); fever and renal involvement encompass the common pentad of symptoms [4, 5]. Unfortunately, TTP is often misdiagnosed or diagnosis is delayed.

TTP results from a severe deficiency of the specific von Willebrand factor (VWF) - cleaving protease, ADAMTS13 (a disintegrin - like and metalloprotease with thrombospondin type 1 motif, member 13) [4, 5]. There are two general forms of ADAMTS13 deficiency, congenital TTP (cTTP) and immune TTP (iTTP). In all TTP patients, the activity of ADAMTS13 in the plasma decreases significantly (< 10%); and patients with iTTP often show the presence of ADAMTS13 inhibitors [5, 6].

Studies have shown that hyperglycemia on admission is associated with a poor prognosis in patients with TTP [7]. A temporary increase in blood glucose can be observed when a relatively severe disease occurs, and stress hyperglycemia has also been found to be associated with increased mortality [8]. In this study, we presented the hospitalization process of 42 patients with TTP in our hospital between 2000 and 2021 and provided a new laboratory basis for clinicians to formulate timely and accurate treatment plans to reduce mortality.

## Materials and methods

### Patient data

**Fig. 1:**
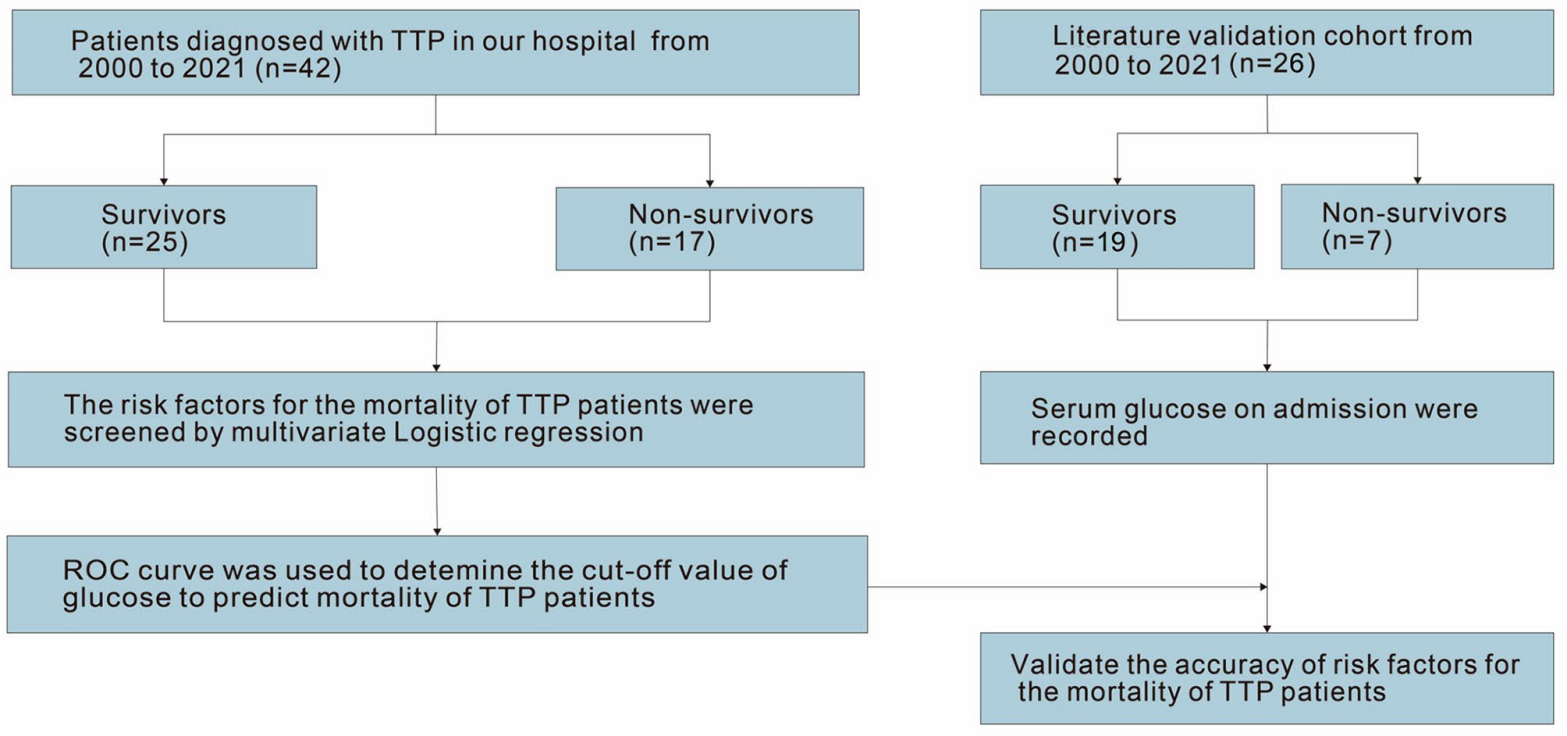
Patient diagnosis and treatment flow. Between 2000 and 2021, 42 patients with TTP in our hospital were retrospectively enrolled, they were defined as survivors (25 cases) and non-survivors (17 cases) according to their survival status. A total of 26 TTP cases with serum glucose results on admission from published literature were collected as the external validation cohort, including 19 cases (73%) of survivors and 7 cases (27%) of non-survivors

The research related to human use has been complied with all the relevant national regulations, institutional policies and in accordance the tenets of the Helsinki Declaration, and has been approved by the authors’ institutional review board or equivalent committee. We enrolled 42 patients who were first diagnosed with TTP in our hospital between 2000 and 2021. The patients were divided into two groups: survivors (25, 60%) and non-survivors (17, 40%), depending on the 30-day post-admission outcome, and then created an external validation cohort with comparable data with the study group to verify the impact of blood glucose level at admission on the mortality of TTP patients. We searched case reports in a Chinese medical full-text and PubMed databases using the search term “thrombotic thrombocytopenic purpura” between 2000 and 2021. A total of 26 patients with TTP with blood glucose results at admission were included (19 survivors [73%] and 7 non-survivors [27%]) (Fig. 1, Supplementary Table S1). In our medical center, the treatment for all TTP patients is according to guidelines and expert consensus, including plasma exchange, glucocorticoid therapy, plasma infusion, red blood cell infusion strategies [9–11], and management of stress hyperglycemia [12].

The diagnostic criteria for TTP were: (a) clinical manifestations: MAHA and thrombocytopenia; (b) blood and biochemical changes: anemia, significantly decreased platelet count, red cell fragmentation > 1% in a peripheral blood smear and significantly elevated lactate dehydrogenase (LDH) serum levels; (c) a pathology consistent with TTP and response to plasma exchange therapy and (d) After 2013, when ADAMTS13 activity measurement was available, ADAMTS13 activity < 10% was also included as one diagnostic criterion [13]. We excluded patients with other thrombotic microangiopathies, such as atypical hemolytic uremic syndrome or transplant-related microangiopathy [9, 10, 14].

We collected data on the following: infections, hypertension, diabetes, connective tissue diseases, and cardiovascular diseases. We also collected laboratory data, including complete blood counts, serum biochemistry, and immunity. In addition, results of ADAMTS13 activity and inhibitor tests available for admitted patients after 2013 were collected. Among the 42 TTP patients, 23 patients received medication before admission, including glucocorticoids (9 cases), antibiotics (8 cases), omeprazole (7 cases), hemostatic drugs (5 cases), traditional Chinese medicine (3 cases), antipyretics (2 cases), antidiarrheals (1 case), and none of them received plasma exchange.

Each patient provided the first fasting blood sample after admission to the hospital. Samples of 2 ml were mixed with EDTA-K_2_ and an anticoagulant for a complete blood count analysis by a Sysmex routine blood analyzer XE2100 (Sysmex, Japan). Samples of 3 ml were centrifuged at 3500 rpm for 5 min to obtain serum samples for biochemical indicators by an AU5800 automatic biochemical analyzer (Beckman, USA) and antinuclear antibody levels were determined by indirect immunofluorescence. Samples of 2.7 ml were mixed with sodium citrate anticoagulant and centrifuged at 3500 rpm for 5 min to obtain plasma samples for ADAMTS13 activity assay, which was performed using residual collagen-binding assays (R-CBA). Samples for antinuclear antibody (stored at 2–8℃) and ADAMTS13 activity and its inhibitor (stored at -30℃ and analyzed by the third-party company) were examined within 48 h, and the rest samples were detected within 2–4 h after sampling. The patient’s glucose level was determined using the glucose oxidase method by serum, which was separated from samples that were centrifuged within half an hour after receipt.

### Statistical analysis

Statistical analysis was performed using SPSS 26.0, and graphs were plotted using GraphPad Prism8 software and R (version 4.1.3). G*Power (version 3.1.9.7) was used for sample size and power estimation. Continuous variables are presented as median (P25 - P75), and the Mann - Whitney U test was used for comparisons between the two groups. Categorical variables are expressed as numbers (percentages), and the chi-square test was used to compare the groups. Multivariate logistic regression analysis was used to screen for risk factors. The ROC curve was used to determine the cut-off value of laboratory indicators to predict mortality. The correlation coefficient was obtained by spearman correlation analysis. Locally weighted scatterplot smoothing (LOWESS) was used to compare the dynamic changes in laboratory indices during hospitalization. Statistical significance was set at *P* < 0.05.

## Results

### Patient characteristics

**Fig. 2:**
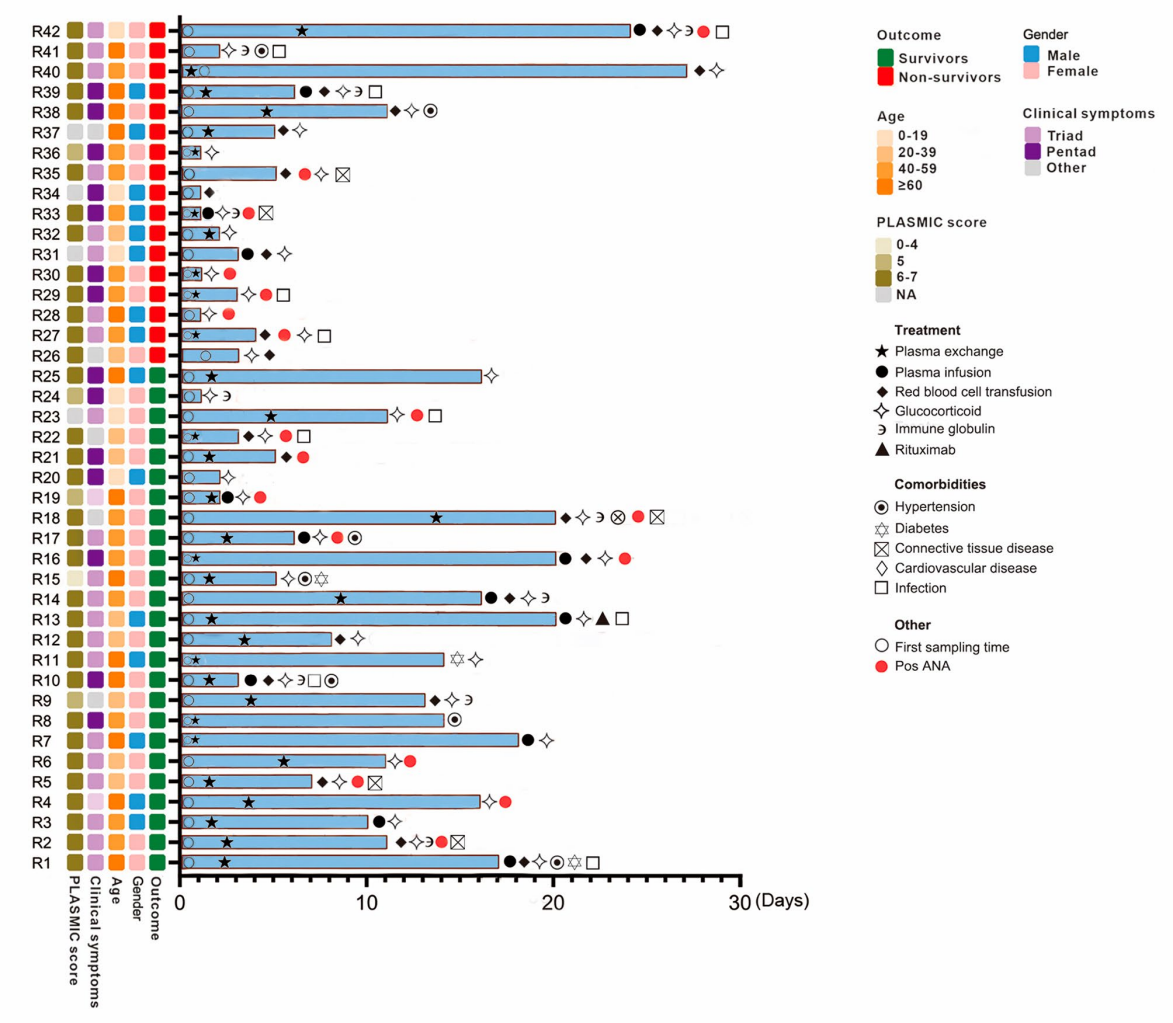
Clinical information, diagnosis and treatment data of patients. The x-axis shows the length of stay, and each row shows information for each patient. The length of stay of the patients is shown with blue bars. The first plasma exchange and sampling time are shown on the blue bar. Comorbidities and other treatments (plasma infusion, glucocorticoid, etc.) are displayed beside the blue bar. Pos ANA: positive antinuclear antibody test

The study cohort of patients with TTP included 25 survivors (18 females and 7 males) and 17 non-survivors (9 females and 8 males), aged 47 (26–65) years and 55 (33–62) years, respectively. All patients’ longitudinal clinical information, diagnosis, treatment, disease progression, and prognosis are comprehensively presented (Fig. 2). We calculated the mortality rates of TTP patients by years (Supplementary Table S2).

### Comparison of clinical and laboratory data

There is a statistical difference in blood glucose on admission between survivors and non-survivors in the whole cohort study with TTP patients (*P* = 0.000) and in the cohort between 2013 and 2021 with available ADAMTS13 data cases (*P* = 0.027). Besides, compared with survivors, in the whole cohort, non-survivors had a shorter length of stay (LOS), lower red blood cells (RBC) counts, higher aspartate aminotransferase (AST) levels and Lactate dehydrogenase (LDH) levels. In the cohort between 2013 and 2021, platelet counts (PLT) of non-survivors were lower than survivors (Table 1).

**Table 1:** Clinical information and laboratory data of the cohort

	2000–2021 (Year)				2013–2021 (Year)			
	Survivors	Non-survivors	*P*		Survivors	Non-survivors	*P*	
Number of patients	25	17			14	9		
Clinical information								
Age	47 (26–65)	55 (33–62)	0.811		54 (32–67)	49 (37–61)	0.659	
Gender			0.206				0.648	
Female, n (%)	18 (75)	9 (53)			8 (57)	6 (67)		
Male, n (%)	7 (25)	8 (47)			6 (43)	3 (33)		
Onset time (Days)	4 (2–7)	6 (3–10)	0.262		2 (2–5)	5 (2–9)	0.306	
Onset to plasma therapy (Days)	7 (5–10)	11 (5–15)	0.135		5 (4–8)	11 (4–14)	0.319	
Neurological symptoms to plasma therapy (Days)	6 (4–9)	11 (4–15)	0.202		5 (4–8)	11 (4–14)	0.319	
Pentad, n (%)	7 (28)	7 (41)	0.374		3 (21)	3 (33)	0.526	
LOS (Days)	11 (5–16)	3 (1–9)	**0.006**		12 (5–17)	4 (2–18)	0.206	
PLASMIC score (6–7), n (%)	20 (83)	13 (93)	0.402		13 (93)	9 (100)	0.412	
Comorbidities								
Diabetes, n (%)	3 (12)	0 (0)	0.138		3 (21)	0 (0)	0.136	
Hypertension, n (%)	3 (12)	2 (11.8)	0.982		3 (21)	2 (22)	0.964	
Connective tissue disease, n (%)	3 (12)	2 (12)	0.982		1 (7)	2 (22)	0.295	
Cardiovascular disease, n (%)	1 (4)	0 (0)	0.404		1 (7)	0 (0)	0.412	
Infection, n (%)	5 (20)	5 (29)	0.482		3 (21)	3 (33)	0.526	
Treatment								
Drugs preuse, n (%)	11 (44)	12 (71)	0.089		7 (50)	4 (44)	0.795	
Number of plasma exchanges	4 (2–5)	1 (0–4)	0.070		4 (2–5)	2 (5–9)	0.485	
Laboratory data								
Pos ANA, n (%)	11 (55)	7 (50)	0.744		5 (42)	5 (56)	0.528	
WBC (×10^9^/L)	9.1 (6.4–9.4)	8.0 (6.4–12.4)	0.929		9.1 (6.4–9.3)	6.9 (5.2–8.9)	0.186	
RBC (×10^12^/L)	2.64 (2.21–2.95)	2.09 (1.79–2.57)	**0.032**		2.81 (2.13–3.01)	2.43 (1.95–2.95)	0.450	
Hb (g/L)	82 (69–90)	69 (57–82)	0.093		84 (70–91)	73 (63–89)	0.488	
PLT (×10^9^/L)	14 (8–24)	8 (7–28)	0.362		13 (9–19)	7 (4–12)	**0.043**	
ALT(U/L)	23 (18–35)	27 (19–38)	0.385		21 (16–36)	28 (19–38)	0.508	
AST(U/L)	50 (34–64)	60 (53–94)	**0.044**		45 (33–63)	57 (52–65)	0.130	
IBIL(µmol/L)	30 (21–46)	45 (36–57)	0.050		30 (17–47)	45 (38–68)	0.068	
Glucose (mmol/L)	6.6 (5.5–8.1)	9.8 (7.1–12.1)	**0.000**		7.5 (5.7–8.4)	9.7 (6.4–11.8)	**0.027**	
LDH (U/L)	887 (563–1300)	1351(1082–1711)	**0.008**		956 (616–1310)	1189 (1026–1387)	0.073	

LOS: Length of stay; Pos ANA: Positive antinuclear antibody test; WBC: White blood cells; RBC: Red blood cells; Hb: Hemoglobin; PLT: Platelet counts; ALT: Alanine aminotransferase; AST: Aspartate aminotransferase; IBIL: Indirect bilirubin; LDH: Lactate dehydrogenase. Onset time: Interval days from clinical symptoms onset to admission. Data are presented as numbers (percentages) or medians (P25-P75). *P* values were obtained by the Mann-Whitney U test or the chi-square test

### TTP risk factor analysis

**Fig. 3:**
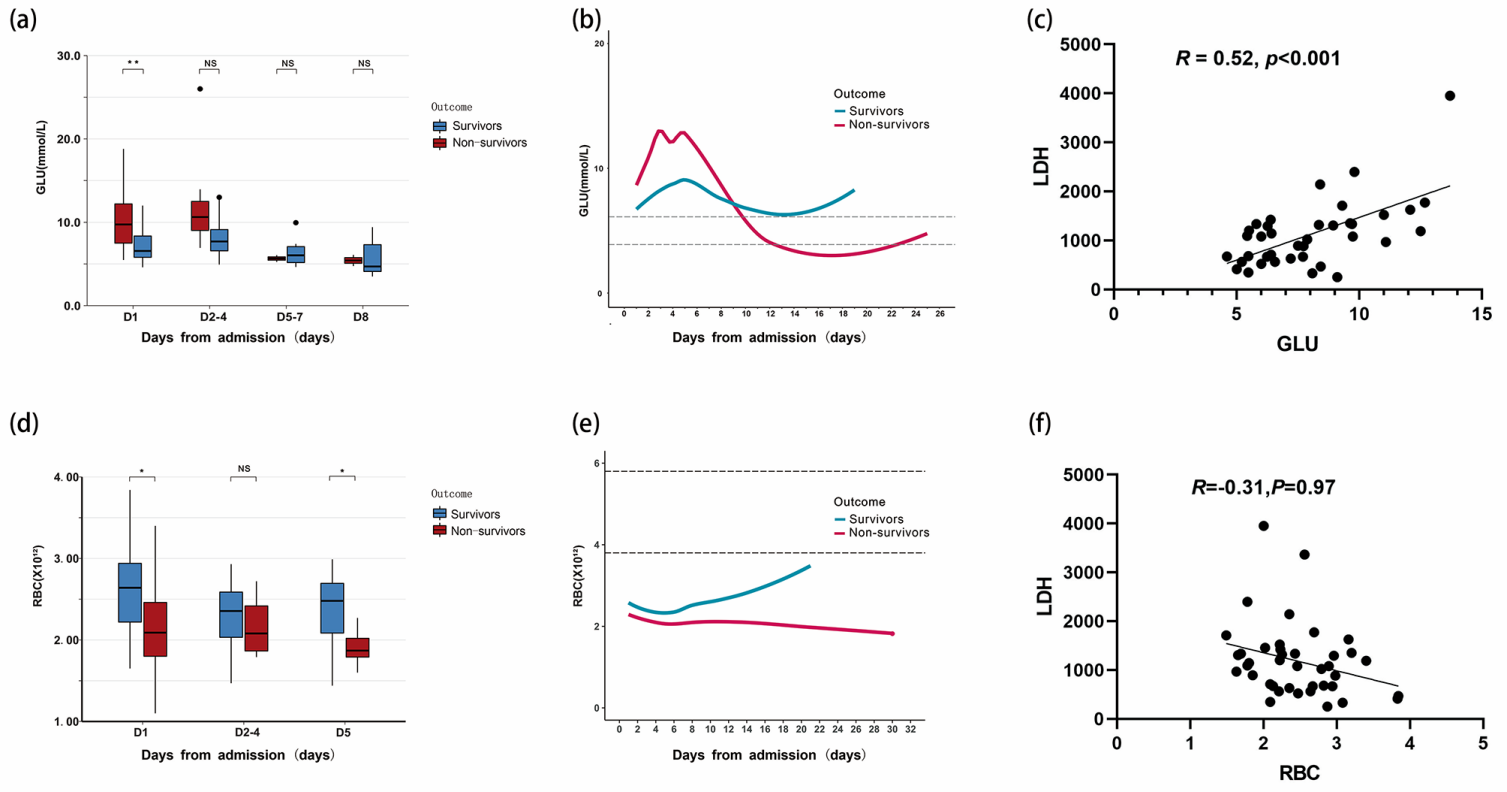
TTP risk factors and blood test dynamics during hospitalization. **(a)** Boxplots of blood glucose according to time distribution. **(b) **LOWESS chart of blood glucose levels during hospitalization. **(c)** Scatter plots depicting the relationship between glucose and LDH. **(d)** Boxplots of RBC according to time distribution. **(e) **LOWESS chart of RBC during hospitalization. **(f) **Scatter plots depicting the relationship between RBC and LDH. RBC: Red blood cells; LDH: lactate dehydrogenase. The correlation coefficient was obtained by spearman correlation analysis. * and ** indicate the *P* values of < 0.05, < 0.01, respectively; NS stands for no statistical difference

In the whole cohort study, after adjusting age and gender, serum glucose levels and RBC were independent predictors of mortality in patients with TTP (glucose: odds ratio [OR], 2.476 [95% confidence interval (CI) 1.368–4.484]; RBC: OR, 0.095 [95% CI 0.011–0.799]). Glucose was significantly correlated with serum LDH (*R* = 0.52, *P* < 0.001), however, RBC was not. There was also no significant difference in blood glucose between patients who received glucocorticoid treatment or not before admission (Supplementary Figure S). The blood glucose levels of survivors during hospitalization are more stable than non-survivors. (Table 2; Fig. 3).

**Table 2:** Risk factors for the mortality of TTP patients (*n* = 42)

	Univariate regression			Multivariate regression	
Index	OR (95% CI)	*P*		OR (95% CI)	*P*
RBC	0.279 (0.082–0.957)	**0.042**		0.095 (0.011–0.799)	**0.030**
Glucose	1.797 (1.233–2.617)	**0.002**		2.476 (1.368–4.484)	**0.003**
LDH	1.001 (1.000–1.002)	0.075		-	-
AST	1.011 (0.995–1.027)	0.164		-	-
Age	1.004 (0.974–1.035)	0.788		0.961 (0.912–1.012)	0.129
Gender	2.286 (0.628–8.320)	0.210		3.139 (0.464–21.223)	0.241

RBC: Red blood cells; LDH: Lactate dehydrogenase; AST: Aspartate aminotransferase

### Glucose and RBC ROC curve mortality predictions

**Fig. 4:**
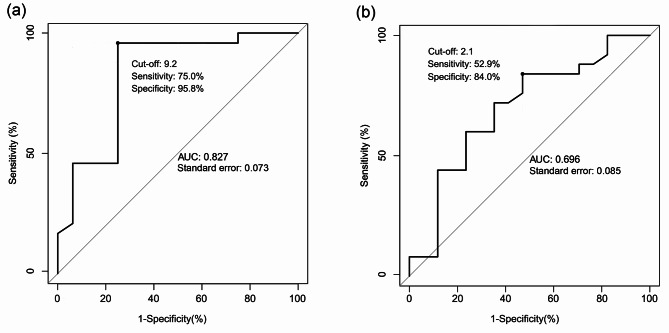
ROC curves for predicting mortality in patients with thrombotic thrombocytopenic purpura. **(A)** ROC curve of GLU; **(B) **ROC curve of RBC. GLU: blood glucose; RBC: red blood cells

The area under the curve (AUC) of glucose for predicting mortality in patients with TTP was 0.827 (cut-off: 9.2 mmol/L, sensitivity, 75.0%; and specificity, 95.8%). The AUC of RBC was 0.696 (cut-off: 2.1 × 10^12^/L, sensitivity: 52.9%, and specificity: 84.0%) (Fig. 4). The external validation group showed 71% and 84% sensitivity and specificity, respectively **(**cut-off: 9.2 mmol/L).

### Blood test changes during hospitalization

**Fig. 5:**
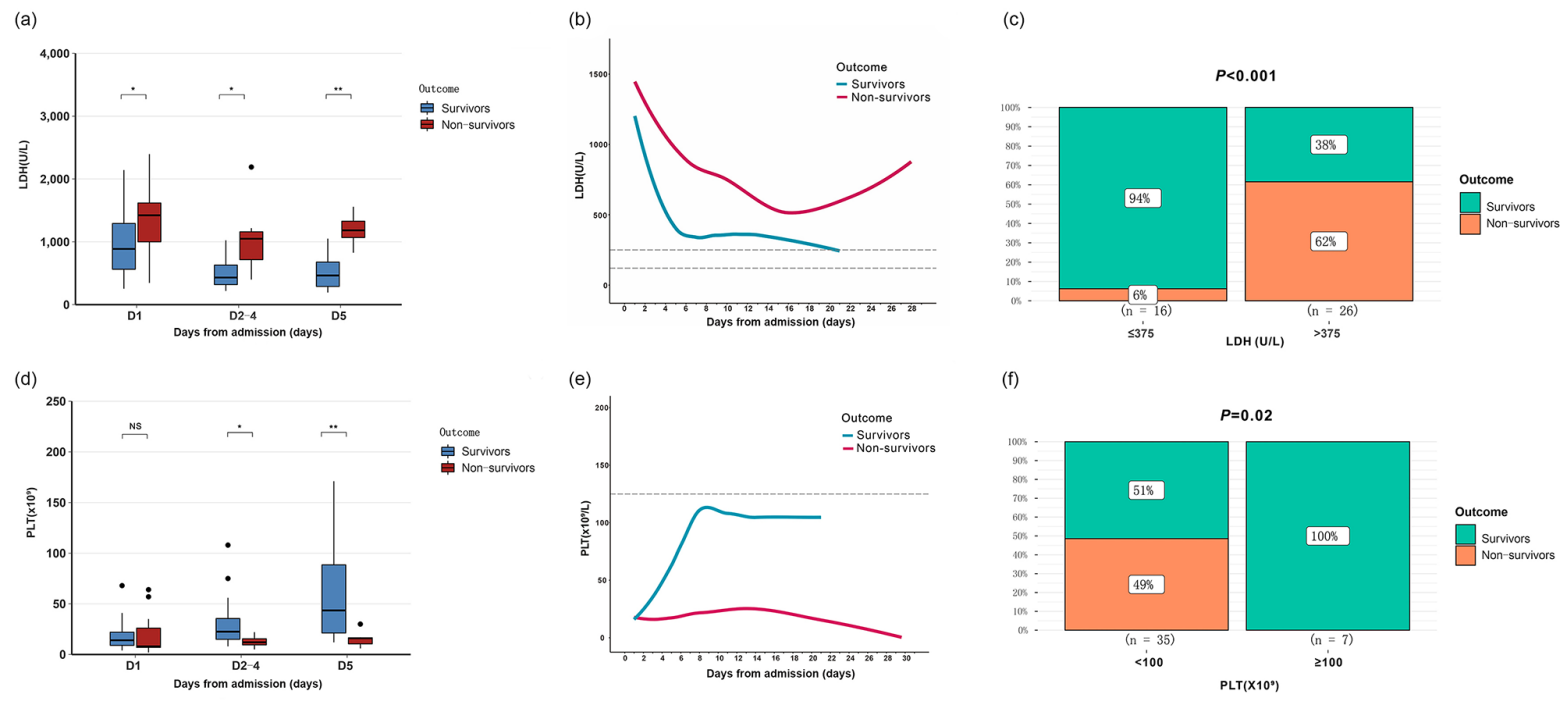
PLT and LDH level changes between survivors and non-survivors. **(a)** Boxplots of LDH according to time. **(b)** LOWESS chart of LDH during hospitalization. **(c)** Histograms of LDH levels on the 5th day of admission. **(d)** Boxplots of PLT according to time distribution.** (e) **LOWESS chart of PLT during hospitalization. **(f)** Histograms of platelet counts on the 5th day of admission. LDH: lactate dehydrogenase; PLT: platelet counts. * and ** indicate the *P* values of < 0.05, < 0.01, respectively; NS stands for no statistical difference

There were significant differences in PLT and LDH levels between survivors and non-survivors by the fifth day of treatment. The prognosis would be better if the patients’ platelet counts could rise to 100 × 10^9^/L (*P* = 0.02) or the serum LDH level fell below 375 U/L (1.5-fold of the normal range of LDH) (*P* < 0.001) after 5 days of treatment (Fig. 5).

## Discussion

TTP is a rare but potentially life-threatening multisystemic disorder [15]. The research process in this study is challenging and very meaningful, for we comprehensively showed the whole course of diagnosis and treatment of all patients, and the correlations between the dynamic changes of laboratory indicators and outcomes were also analyzed. Decades ago, the diagnosis of TTP mainly depended on the clinical manifestations of patients. From 2001 to 2005, the diagnosis of TTP mainly relied on the clinical manifestations of patients [9], resulting in a high mortality rate. With improved diagnostic awareness, ADAMTS13 activity and its inhibitor testing, and the use of rituximab for TTP patients, from 2006 to 2021, the mortality rate was lower than that of 2001 to 2005, but no downward trend was observed year by year, which means there is no obvious “time effect” on mortality in this cohort. Therefore, it is necessary to seek new laboratory biomarkers to identify high-risk patients to reduce TTP patients’ mortality rate.

However, at least one of the ten TTP patients may still cause fatal consequences [3]. Indicators for early prediction of hospitalization outcomes can guide clinical practice to improve the prognosis of patients through specific interventions, thus reducing mortality. In this study, we collected clinical information and laboratory results of all patients with TTP. We found that glucose levels and RBC counts were independent predictors of mortality in patients with TTP, and there were also significant differences in blood glucose levels between survivors and non-survivors in cohorts with available ADAMTS13 data. However, hemoglobin had no significant difference between the survival and non-survival groups. We speculate that TTP patients exhibit microangiopathic hemolytic anemia, characterized by mechanical damage to RBC by microthrombus [10]. Therefore, the decrease in RBC may be earlier than that of hemoglobin. Additionally, glucose concentration was significantly correlated with LDH. Simultaneously, we also collected published studies with the same period of the study cohort as the external validation cohort to prove that the blood glucose level at admission can predict the mortality of TTP patients.

TTP is associated with infections, diabetes, hypertension, obesity, metabolic syndrome, and cardiovascular disease [6]; the incidence of cardiovascular complications in patients hospitalized for TTP is approximately 25% and is associated with higher mortality [16]. However, other reports have indicated that there was no difference between the survivors and non-survivors with diabetes, hypertension, and obesity [14]. In this cohort study, infection and connective tissue disease were the two most common trigger factors for TTP, with an incidence rate of 24% and 12%, respectively, which were similar to other studies for the same diseases [14, 17]. However, there were no differences between the survivors and non-survivors.

TTP mortality-related laboratory indicators, including anti-ADAMTS13 antibody [18], high LDH [18] and troponin I [19], low serum total protein or albumin, and extended activated partial thromboplastin time have been reported [20]. Hai-Xu Wang et al. [7] reported that high blood glucose level was associated with a poor prognosis in patients with TTP. Our study is one of the first to discuss the cut-off value of blood glucose for mortality in detail.

We found that blood glucose levels between survivors and non-survivors differed on admission and in their dynamic changes during hospitalization. Blood glucose levels in non-survivors were significantly higher than those in survivors on admission, with a wider range of fluctuations during hospitalization. The survivors were close to the upper limit of the reference range at admission and showed a slow upward trend. After day 8, the survivors showed a downward trend in glucose levels and remained near the upper limit of the reference range, which was relatively stable. We show that high blood glucose levels at admission and blood glucose fluctuation widely during hospitalization are risk mortality-related factor in TTP patients. Previous studies have also shown that a high variation in blood glucose levels is an independent predictor of mortality in critically ill patients [21].

Elevated glucose may be caused by poor control of chronic hyperglycemia disease or the stress state of the disease. And stress hyperglycemia usually occurs in relatively serious diseases [22, 23]. The American Diabetes Association and the American Association of Clinical Endocrinologists define stress hyperglycemia or hospital-related hyperglycemia as any blood glucose concentration > 140 mg/dL (> 7.8 mmol/L) in patients without a prior history of diabetes [12]. The non-survivors in this study cohort had no history of diabetes. Some scholars found that in nondiabetic patients, the existence of acute hyperglycemia can lead to poor long-term prognosis and an increase in in-hospital mortality [24]. The cut-off value of blood glucose in this study was 9.2 mmol/L, which is higher than 7.8 mmol/L.

Several studies [25, 26] have shown that metabolic syndrome and obesity cause different degrees of damage to the endocrine system and affect survival because of their connection with elevated blood glucose levels. Among the 42 patients, the BMI index of 35 patients was lower than 30 kg/m^2^, and the other 7 patients did not have their height and weight records because of their critical status at admission, but no one was diagnosed as obese. We have recorded the data of each patient in our study, and found a patient with hypertension and diabetes, and her BMI index was 27.24 kg/m^2^, but her waist circumference was not measured, so the patient is suspicious of metabolic syndrome. Among the other patients, there was one patient with diabetes, four with hypertension, and one with fatty liver, but no patient had more than three characteristics in the diagnostic criteria of metabolic syndrome [27] at the same time.

Impairment of blood glucose homeostasis is common after injury and severe disease; the body releases abundant cortisol hormones such as epinephrine and catecholamines [28]. Stress-related blood glucose elevation can protect the body to a certain extent, but excess blood glucose can be converted into a large amount of free fatty acids, which can exacerbate oxidative stress and endothelial injury and have toxic effects on the myocardium through disruption of calcium homeostasis, myocardial reperfusion injury, and intracellular acidosis [29]. Furthermore, in animal studies, high concentrations of free fatty acids during myocardial ischemia increased the myocardial oxygen demand and decreased myocardial contractility [30]. Many studies have shown that cardiovascular disease is the leading cause of mortality in patients with TTP [14, 16]. Therefore, it can be inferred that hyperglycemia caused by stress may be a mortality-related factor in these patients.

We also compared the changes in other TTP-related blood test results between non-survivors and survivors. LDH levels and PLT counts can be essential indicators for judging the efficacy of TTP treatment and monitoring recurrence [11]. The LDH of the survivors in this study decreased significantly five days after admission and remained close to the upper limit of the reference range, which was consistent with the results reported by Staley et al. [20]. The PLT counts had a significant upward trend after admission, and there was a considerable difference on the 5th day.

This study had some limitations. First, it was a single-center retrospective study with a relatively small sample size due to the low incidence rate of TTP; the power of this study was 0.74. Second, because the outcome of patients with TTP in this study was examined 30 days after admission, there may be bias for some patients with a longer disease course. Third, because the non-survivors were all patients with no history of diabetes, stress-induced hyperglycemia in patients with diabetes was not evaluated, there may be some unknown and unpredictable potential factors in this study. Fourth, ADAMTS13 data was not available for TTP patients before 2013.

## Conclusion

This study found that hyperglycemia at admission and unstable blood glucose levels during hospitalization may be the mortality-related factors in patients with TTP. This provides a new laboratory basis for monitoring the treatment of patients with TTP, improving prognosis, and reducing mortality. In addition, we found that the recovery of platelets and the significant decrease in serum LDH within five days of treatment are related to a good prognosis in TTP patients. We suggest that these two concepts can be used as an early warning index for the mortality stratification of TTP patients.

### Electronic supplementary material

Below is the link to the electronic supplementary material.

Supplementary Material 1 Supplementary figure S. Boxplots of blood glucose concentration between patients with and without glucocorticoid pre-treatment before admission

Supplementary Material 2 Supplementary Table S1 Data of the validation cohort

Supplementary Material 3 Supplementary Table S2 30-day Mortality of TTP patients by years

## Data Availability

The datasets are available from the corresponding author upon reasonable request.
