# Supplementary figures and images for: Stress-induced hyperglycemia is associated with the mortality of thrombotic thrombocytopenic purpura patients
Source: Diabetol Metab Syndr. 2024 Feb 15;16:44. doi: 10.1186/s13098-024-01275-2 (PMC10870494; doi:10.1186/s13098-024-01275-2)

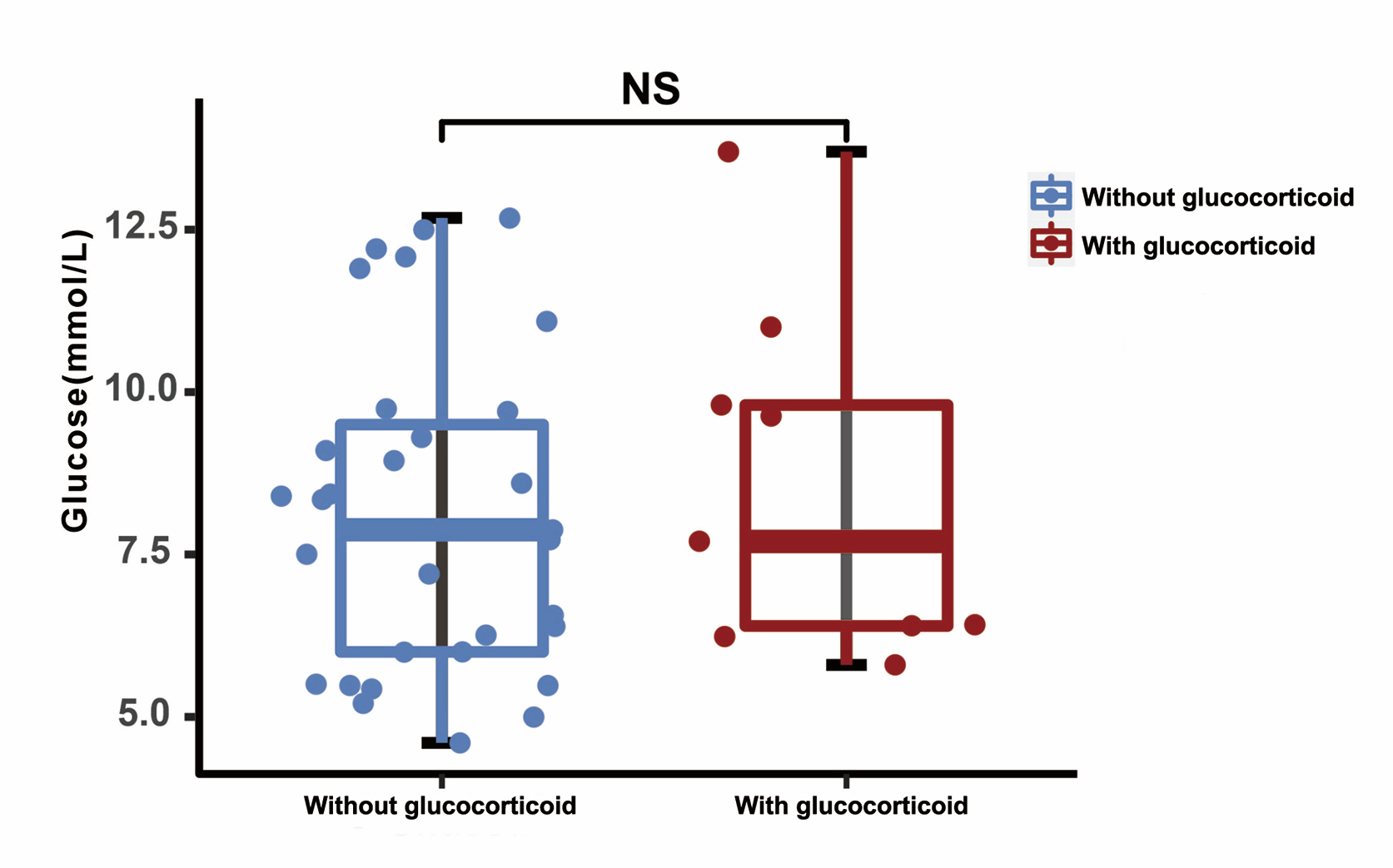

Supplement: Supplementary file 1 — Supplementary Material 1 Supplementary figure S. Boxplots of blood glucose concentration between patients with and without glucocorticoid pre-treatment before admission [file 13098_2024_1275_MOESM1_ESM.tiff]
