# Supplementary material for: Stress-induced hyperglycemia is associated with the mortality of thrombotic thrombocytopenic purpura patients
Source: Diabetol Metab Syndr. 2024 Feb 15;16:44. doi: 10.1186/s13098-024-01275-2 (PMC10870494; doi:10.1186/s13098-024-01275-2)
Supplement: Supplementary file 2 — Supplementary Material 2 Supplementary Table S1 Data of the validation cohort [file 13098_2024_1275_MOESM2_ESM.pdf]

Supplementary Table S1: Data of the validation cohort

Case	Title	Journal	Date-publication	Sex	Age	GLU (mmol/L)	Outcome
1	Comments on forensic dispute cases in medical disputes——Whether there is a causal relationship between the delay in diagnosis of thrombotic thrombocytopenic purpura and the death of patients	Shanghai Medical Journal	2007	Female	/	16.47	Non-Survivor
2	A case report of thrombotic thrombocytopenic purpura with cough and shortness of breath as the first manifestation and literature review	Chongqing Medicine	2021	male	53	14.68	Non-Survivor
3	Analysis of one case of death due to thrombotic thrombocytopenic purpura	World Health Digest	2010	Female	48	17.9	Non-Survivor
4	One case of drug-induced thrombotic thrombocytopenic purpura	Chinese Journal of Leprosy and Skin Diseases	2003	male	46	11.5	Non-Survivor
5	Clinical observation of 3 cases of thrombotic thrombocytopenic purpura treated with plasma exchange combined with hormone	International Journal of Internal Medicine	2009	male	40	10	Non-Survivor
6	A case of malignant tumor-related thrombotic thrombocytopenic purpura	Journal of Cancer Control and Treatment	2010	male	68	5.7	Non-Survivor
7	Misdiagnosis of two cases of thrombotic thrombocytopenic purpura and literature review	Clinical Misdiagnosis & Mistherapy	2013	male	56	7.92	Non-Survivor
8	Clinical observation of 3 cases of thrombotic thrombocytopenic purpura treated with plasma exchange combined with hormone	International Journal of Internal Medicine	2009	Female	27	11	Survivor
9	A case report of Sjögren's syndrome complicated with thrombotic thrombocytopenic purpura	Practical Journal of Clinical Medicine	2014	Female	63	6.08	Survivor
10	A case report of autoimmune hepatitis complicated with thrombotic thrombocytopenic purpura	Journal of Clinical Hepatology	2018	Female	60	5.98	Survivor
11	Plasma exchange in the treatment of a patient with thrombotic thrombocytopenic purpura	International Journal of Blood Transfusion and Hematology	2013	male	19	6.68	Survivor
12	Nonsteroidal Anti-inflammatory Drug Induced Thrombotic Thrombocytopenic Purpura	Clinical Medicine Insights: Blood Disorders	2013	male	21	5.3	Survivor

13	Systemic lupus erythematosus complicated with thrombotic thrombocytopenic purpura: a case and literature review	Shanghai Medical Journal	2005	Female	24	4	Survivor
14	A case report of thrombotic thrombocytopenic purpura	Journal of Youjiang Medical University for Nationalities	2006	male	3.5	6	Survivor
15	A case report of thrombotic thrombocytopenic purpura (TTP) misdiagnosed as encephalitis	Journal of Apoplexy and Nervous Diseases	2008	male	45	8.37	Survivor
16	A case report of thrombotic thrombocytopenic purpura	Journal of Tianjin Medical University	2003	male	51	2.7	Survivor
17	A case of thrombotic thrombocytopenic purpura misdiagnosed as cerebrovascular disease	Journal of Baotou Medical College	2004	Female	50	5.1	Survivor
18	Nursing care of a patient with thrombotic thrombocytopenic purpura treated with plasma exchange combined with hormone therapy	Chinese General Practice Nursing	2017	male	27	8.5	Survivor
19	Acute pancreatitis-induced thrombotic thrombocytopenic purpura with recurrent acute pancreatitis	Clinical Journal of astroenterology	2016	male	26	6.89	Survivor
200	Treatment of Acute Kidney Injury in Hemolythic Uremic Syndrome (TTP)	Medical Archives	2018	Female	39	7.9	Survivor
21	Acute thrombotic thrombocytopenic purpura following orthopedic surgery: a case report	Archives of Orthopaedic and Trauma Surgery	2006	Female	57	12.4	Survivor
22	Case 37-2010: A 16-Year-Old Girl with Confusion, Anemia, and Thrombocytopenia	The New England Journal of Medicine	2010	Female	16	7.5	Survivor
23	Successful Treatment with Cyclosporine of Thrombotic Thrombocytopenic Purpura Refractory to Corticosteroids and Plasma Exchange	Therapeutic Apheresis and Dialysis	2011	male	32	5.94	Survivor
24	A Case of Severe Thrombotic Thrombocytopenic Purpura With Concomitant Legionella Pneumonia:Purpura With Concomitant Legionella Pneumonia:	American Journal of Therapeutics	2011	male	65	10	Survivor
25	Acute pancreatitis induced thrombotic thrombocytopenic purpura	Indian Journal of Crit Care Medicine	2014	male	43	7.3	Survivor

26	Ibuprofen-induced thrombotic thrombocytopenic purpura	American Journal of Emergency Medicine	2016	male	37	6.4	Survivor
