# Supplementary material for: Stress-induced hyperglycemia is associated with the mortality of thrombotic thrombocytopenic purpura patients
Source: Diabetol Metab Syndr. 2024 Feb 15;16:44. doi: 10.1186/s13098-024-01275-2 (PMC10870494; doi:10.1186/s13098-024-01275-2)
Supplement: Supplementary file 3 — Supplementary Material 3 Supplementary Table S2 30-day Mortality of TTP patients by years [file 13098_2024_1275_MOESM3_ESM.pdf]

Supplementary Table S2: 30-day Mortality of TTP patients by years

Year	Non-survivors (n=17)	Total (n=42)	30-day Mortality (%)
2001 - 2005	3	4	75.0
2006 - 2010	4	11	36.4
2011 - 2013	3	8	37.5
2014 - 2016	3	7	42.9
2017 - 2019	3	8	37.5
2020 - 2021	1	4	25.0
